# Supplementary material for: Neutralizing antibody responses to SARS-CoV-2 in symptomatic COVID-19 is persistent and critical for survival
Source: Nat Commun. 2021 May 11;12:2670. doi: 10.1038/s41467-021-22958-8 (PMC8113594; doi:10.1038/s41467-021-22958-8)
Supplement: Supplementary file 1 — Supplementary Information [file 41467_2021_22958_MOESM1_ESM.pdf]

Supplementary Information for

Neutralizing antibody responses to SARS-CoV-2 in symptomatic COVID-19 is persistent and critical for survival.

by Stefania Dispinseri et al.

Supplementary Figures 1 to 10

- Figure 1: Schematic representation of transfer vectors and plasmids.
- Figure 2: Gating strategy used for FACS analysis to detect ACE expression on VEROE6 cells.
- Figure 3: Correlation of anti-SARS-CoV2 spike neutralizing and RBD, S1+S2 antibodies during follow-up of the COVID-19 patients.
- Figure 4: Development and kinetics of SARS-CoV-2 antibody responses of non-hospitalized COVID-19 patients.
- Figure 5: Univariate Hazard Ratios (HR) for time to a negative SARS-CoV-2 viral RNA RT-PCR of the nasopharyngeal swab and to death of 134 hospitalized COVID-19 patients.
- Figure 6: Bivariate Hazard Ratios (HR) for time to death of COVID-19 patients.
- Figure 7: HCoV-HKU1 and -OC43 S2 IgG correlation with neutralization is temporary.
- Figure 8: Variations of the SARS-CoV-2 neutralizing titers between the 2nd and 3rd out-patient visit.
- Figure 9: Gating strategy used for FACS analysis to detect SARS-CoV-2 spike.
- Figure 10. Schematic representation of plasmids used for the LIPS.

Supplementary Tables 1 to 5

- Table 1: Laboratory values at first Biobank blood sampling of the COVID-19 study population.
- Table 2: SARS-CoV-2 Antibody responses according to week from symptoms onset. (provided as xls file)
- Table 3: Characteristics of non-hospitalized COVID-19 patients.
- Table 4: Characteristics of COVID-19 patients grouped according to their SARS-CoV-2 neutralizing antibody response. (provided as xls file)
- Table 5: COVID-BioB study team and collaborators.

Supplementary Figures

Supplementary Figure 1

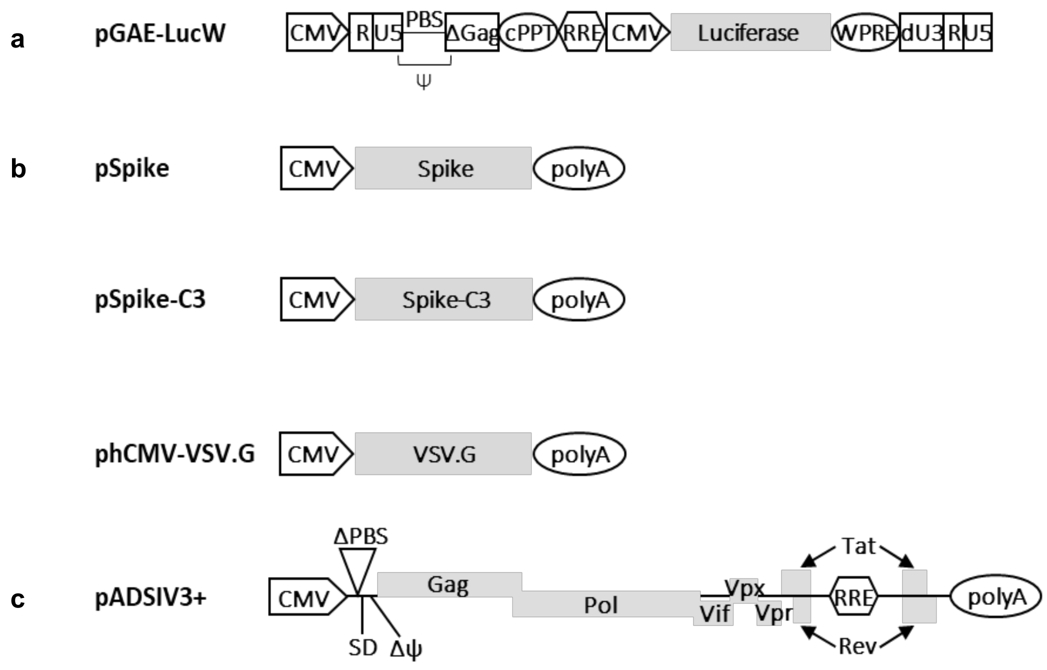

Footnote to Supplementary Figure 1: Schematic representation of transfer vectors and plasmids. (a) the lentiviral transfer vector plasmid expressing luciferase reporter gene under the control of CMV promoter (pGAE-LucW); (b) the pseudotyping plasmids expressing full-length Spike (pSpike), cytoplasmic tail truncated Spike (pSpike-C3) and VSV.G envelope (phCMV-VSV.G); (c) the packaging plasmid providing the proteins for producing the vector particles (pADSIV3+). The packaging signal (ψ), the primer binding site (PBS), the deleted packaging signal (Δψ), the major splice donor (SD), the bovine growth hormone polyadenylation signal (polyA) and the central polypurine tract (cPPT) are indicated.

Supplementary Figure 2

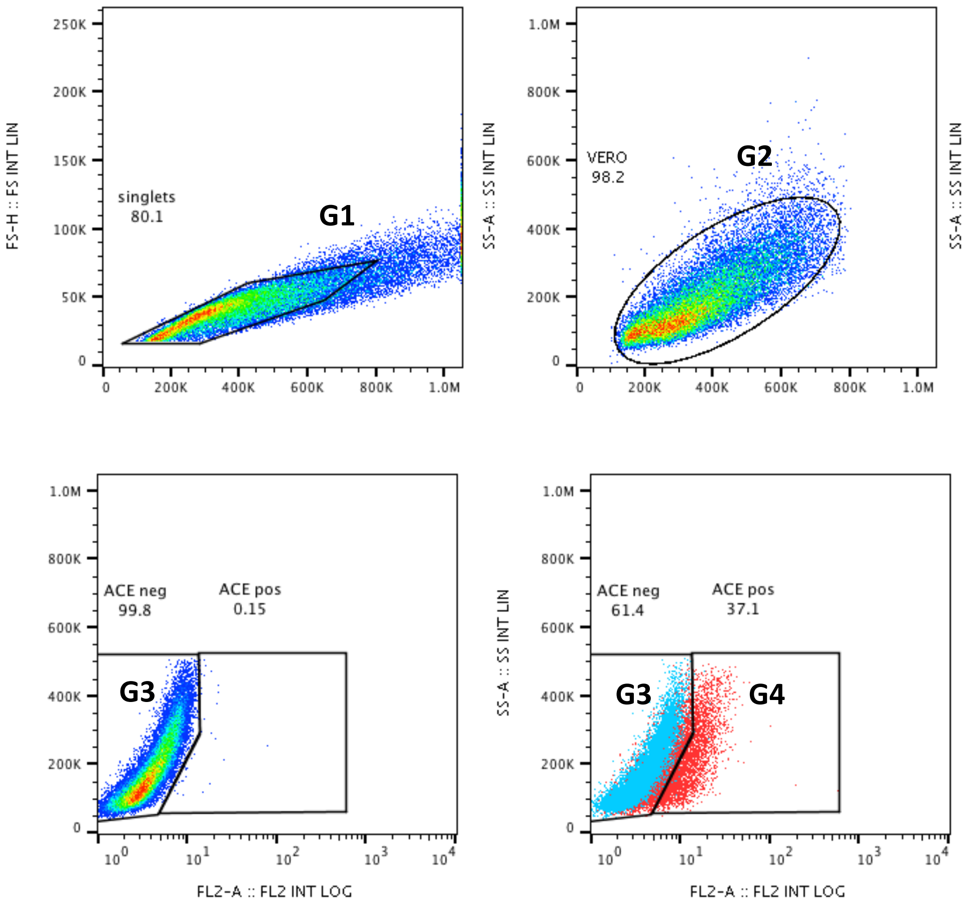

Footnote to Supplementary Figure 2: Gating strategy used for FACS analysis to detect ACE expression on cells. The first gate (G1) was set to include the singlets. Forward (FSC) and side scatter (SSC) gating (G2) was used to identify the cells of interest. The identification of the ACE2 positive cells was performed by using a primary mouse anti-human ACE2 antibody (Millipore, Catalog Number: MAB5676) followed by a secondary goat anti-mouse IgG-PE (SoutherBiotech; Catalog Number: 1030-09). Staining with secondary antibody only (G3) was used as negative control to set the gate of negative cells (G3) and quantify the percentage of positive cells expressing ACE2 (G4).

Supplementary Figure 3

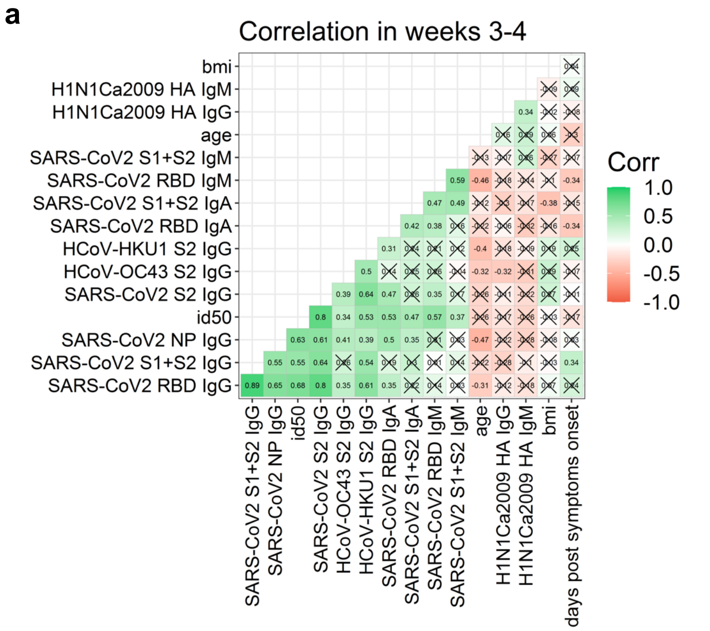

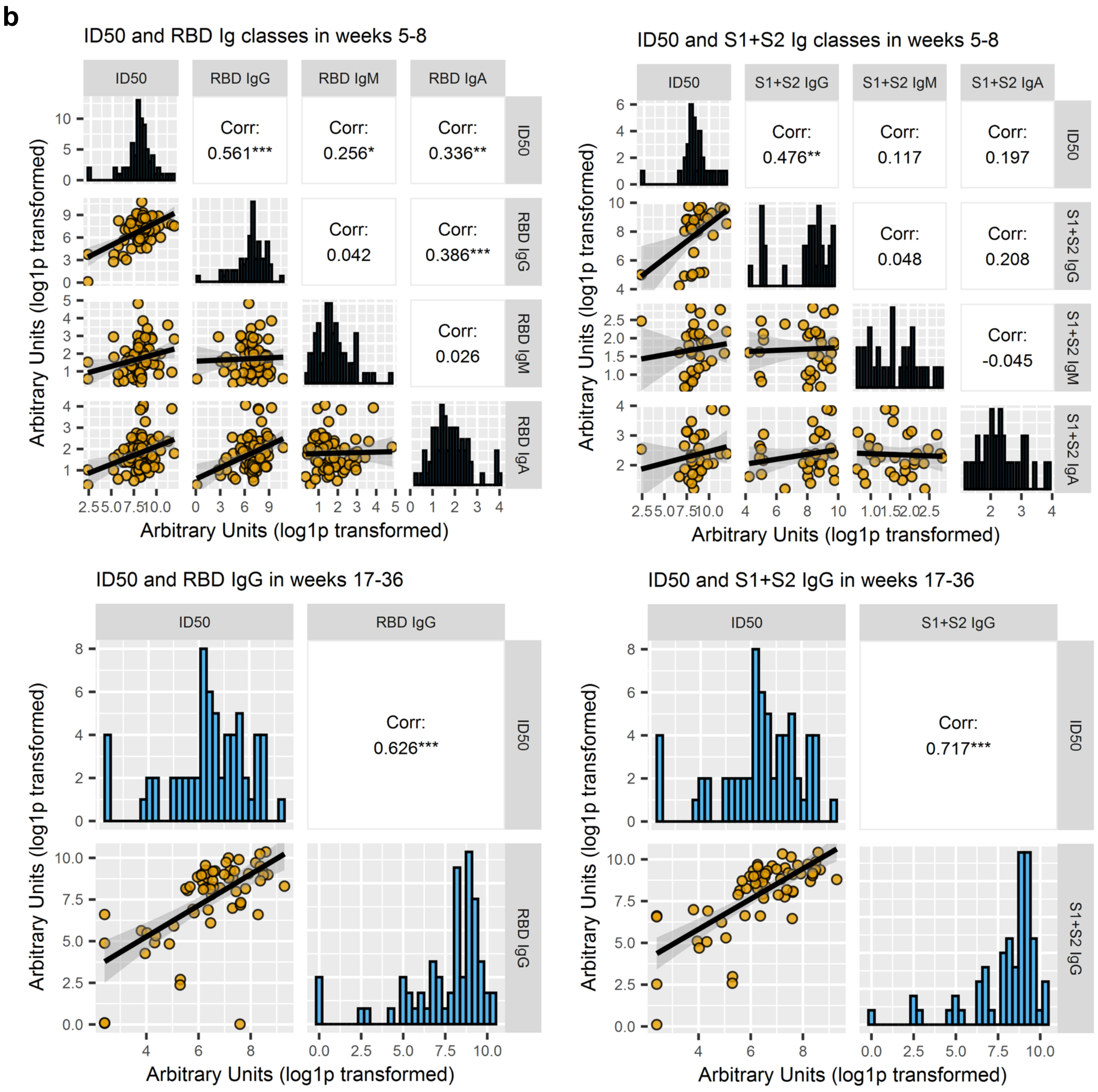

Footnote to Supplementary Figure 3: Correlation of anti-SARS-CoV2 spike neutralizing and RBD, S1+S2 antibodies during follow-up of the COVID-19 patients. Sera of 162 COVID-19 patients, collected at the indicated timepoints from symptoms onset, were assessed by LV-based neutralization assay (ID50) and the LIPS assay to the various antigens, as indicated in the panels. (a) Correlation matrix of the indicated variables at week 3-4. For each pair, the Pearson's correlation coefficient is shown as number and on a color scale. Statistically non-significant correlations are crossed. (b) Sera of COVID-19 patients, collected at the indicated timepoints from symptoms onset, were assessed by LV-based neutralization assay (ID50) and the LIPS assay, indicated in grey labels above each row/column. Boxes under the diagonal show each correlation plot of the ID50 reciprocal and arbitrary units after log10 conversion. Dots correspond to individual measurements; the black line represents the regression line and the grey area its 95%CI. Boxes on the diagonal show as histograms the distribution of values in each assay. Boxes above the diagonal show the corresponding Pearson correlation analysis coefficients. Asterisks correspond to the following p values: *** $p \leq 0.001$; ** $p \leq 0.01$; * $p \leq 0.05$. Source data are provided as a Source Data file.

Supplementary Figure 4

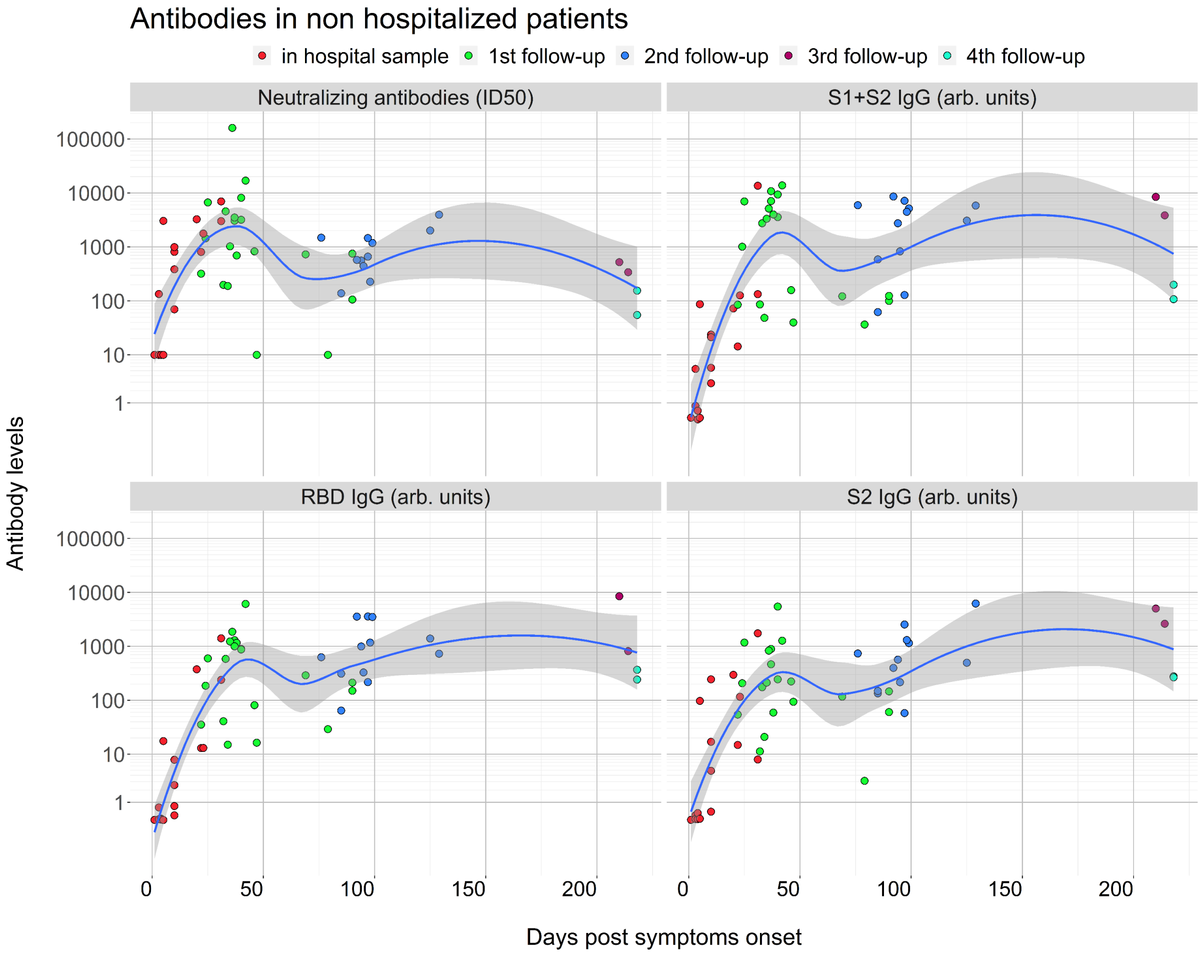

Footnote to Supplementary Figure 4: Development and kinetics of SARS-CoV-2 antibody responses of non-hospitalized COVID-19 patients. Each colored dot corresponds to the ID50 reciprocal of a given infected individual's serum of 26 patients. Shown is the moving average of data + SE (black curve line + grey band) as obtained by a LOESS curve fitting polynomial regression. Sampling occurred during hospital attendance (ER or ward), and at follow-up visits post discharge; colors define the number of the visits attended. In Table 1 the serum sample availability of the non-hospitalized COVID-19 patients is described. The clinical characteristics are described in Supplementary Table 3. Source data are provided as a Source Data file.

Supplementary Figure 5

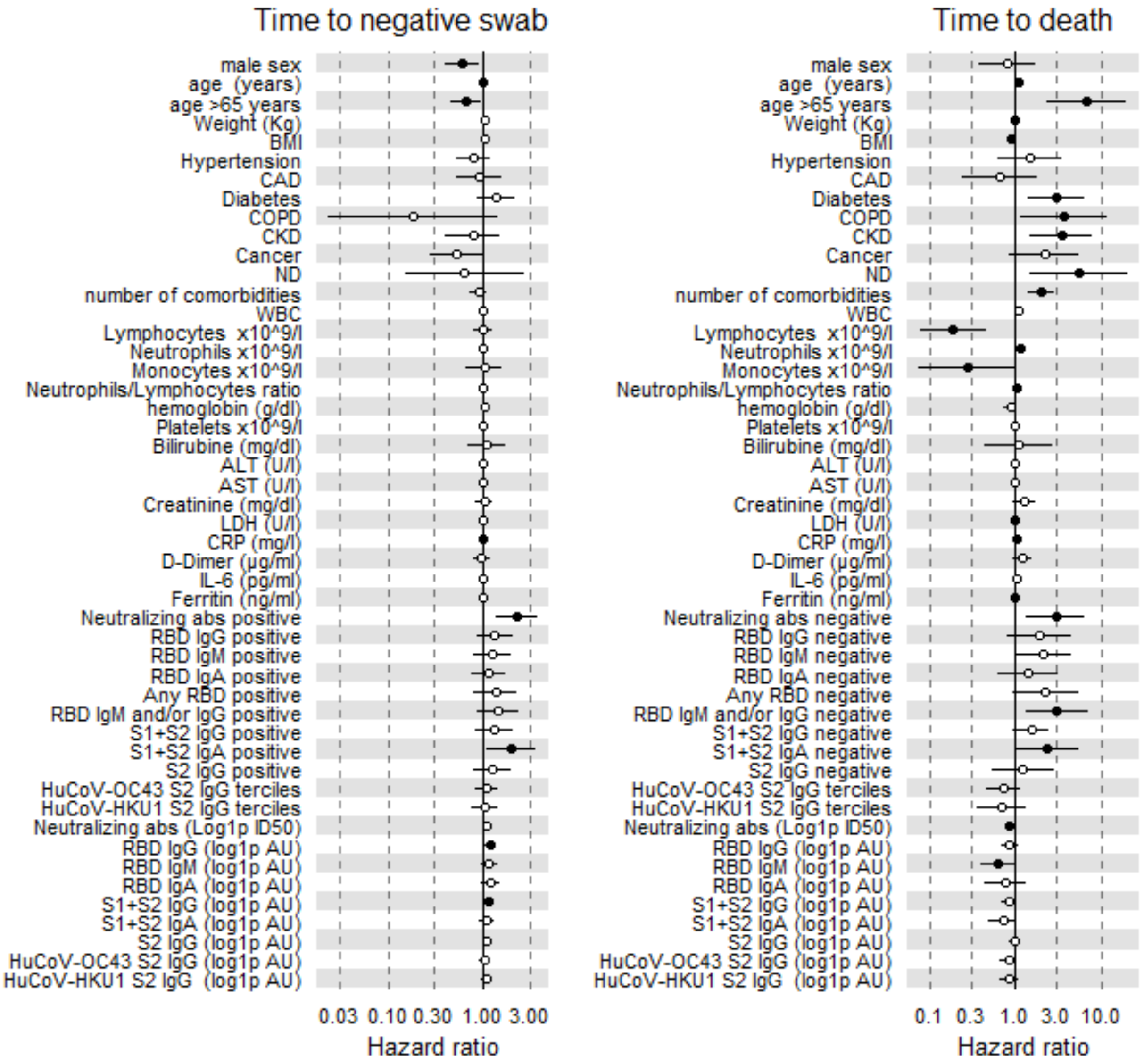

Footnote to Supplementary Figure 5: Univariate Hazard Ratios (HR) for time to a negative SARS-CoV-2 viral RNA RT-PCR of the nasopharyngeal swab and to death of 134 hospitalized COVID-19 patients. Forest plots of Hazard Ratios obtained by univariable Cox regression analysis of the shown variables at the time of the in-hospital serum sampling. The analysis was adjusted for sex and age. Dots represent the HR, filled dots stand for $p<0.05$ (two-sided). Wald statistics were used for comparison. Abbreviations are: Body Mass Index (BMI), Coronary Artery Diseases (CAD), Chronic obstructive pulmonary disease (COPD), Chronic Kidney Disease (CKD), Neurodegenerative disease (ND), White Blood cells (WBC), Lactate dehydrogenase (LDH), C-reactive protein (CRP). Antibody binding are to SARS-CoV-2, when not otherwise specified. Antibody binding is expressed in arbitrary units (AU). Source data are provided as a Source Data file.

Supplementary Figure 6

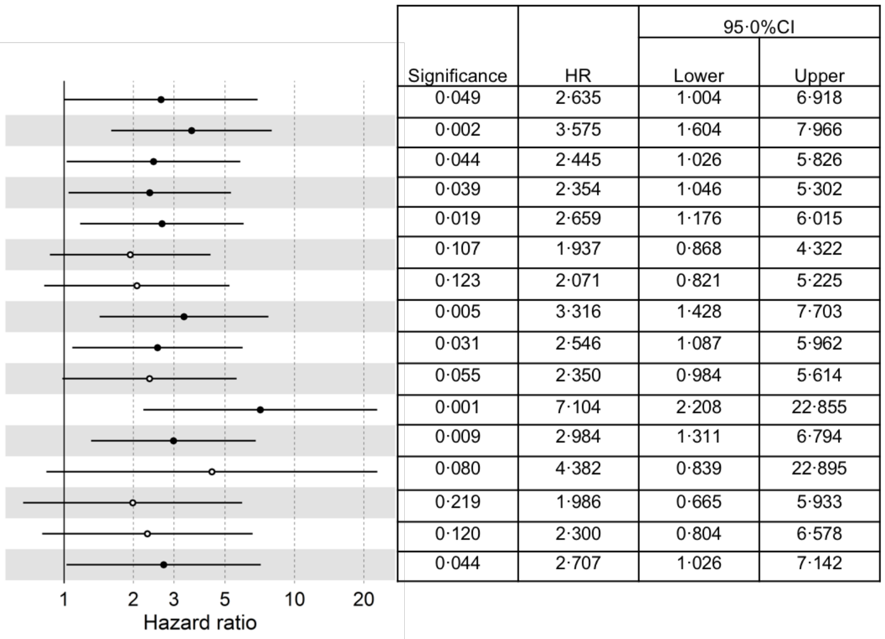

	Significance	HR	95·0%CI Lower	95·0%CI Upper
BMI	0·049	2·635	1·004	6·918
Diabetes	0·002	3·575	1·604	7·966
COPD	0·044	2·445	1·026	5·826
CKD	0·039	2·354	1·046	5·302
ND	0·019	2·659	1·176	6·015
number of comorbidities	0·107	1·937	0·868	4·322
Lymphocytes x10^9/l	0·123	2·071	0·821	5·225
Neutrophils x10^9/l	0·005	3·316	1·428	7·703
Monocytes x10^9/l	0·031	2·546	1·087	5·962
Neutrophils/Lymphocytes ratio	0·055	2·350	0·984	5·614
LDH (U/l)	0·001	7·104	2·208	22·855
CRP (mg/l)	0·009	2·984	1·311	6·794
Ferritin (ng/ml)	0·080	4·382	0·839	22·895
RBD IgM or IgG negative	0·219	1·986	0·665	5·933
RBD IgM AU	0·120	2·300	0·804	6·578
S1+S2 IgA negative	0·044	2·707	1·026	7·142

Footnote to Supplementary Figure 6: Bivariate Hazard Ratios (HR) for time to death of COVID-19 patients. Forest plot of Hazard Ratios (HR) with lower and upper limit of the 95%CI for neutralizing antibodies at the time of in-hospital serum sampling and survival in COVID-19 patients calculated with multivariable Cox regression analysis. The analysis used a neutralization negative score, corrected for age and sex, and the shown variables measured at the time of in-hospital serum sampling. Dots represent the HR, filled dots stand for $p<0.05$ (two-sided). Wald statistics were used for comparison. Abbreviations are: Body Mass Index (BMI), Coronary Artery Diseases (CAD), Chronic obstructive pulmonary disease (COPD), Chronic Kidney Disease (CKD), Neurodegenerative disease (ND), Lactate dehydrogenase (LDH), C-reactive protein (CRP). Antibody binding (IgG, IgM and IgA) are to SARS-CoV-2, and expressed in arbitrary units (AU)**.** Source data are provided as a Source Data file.

Supplementary Figure 7

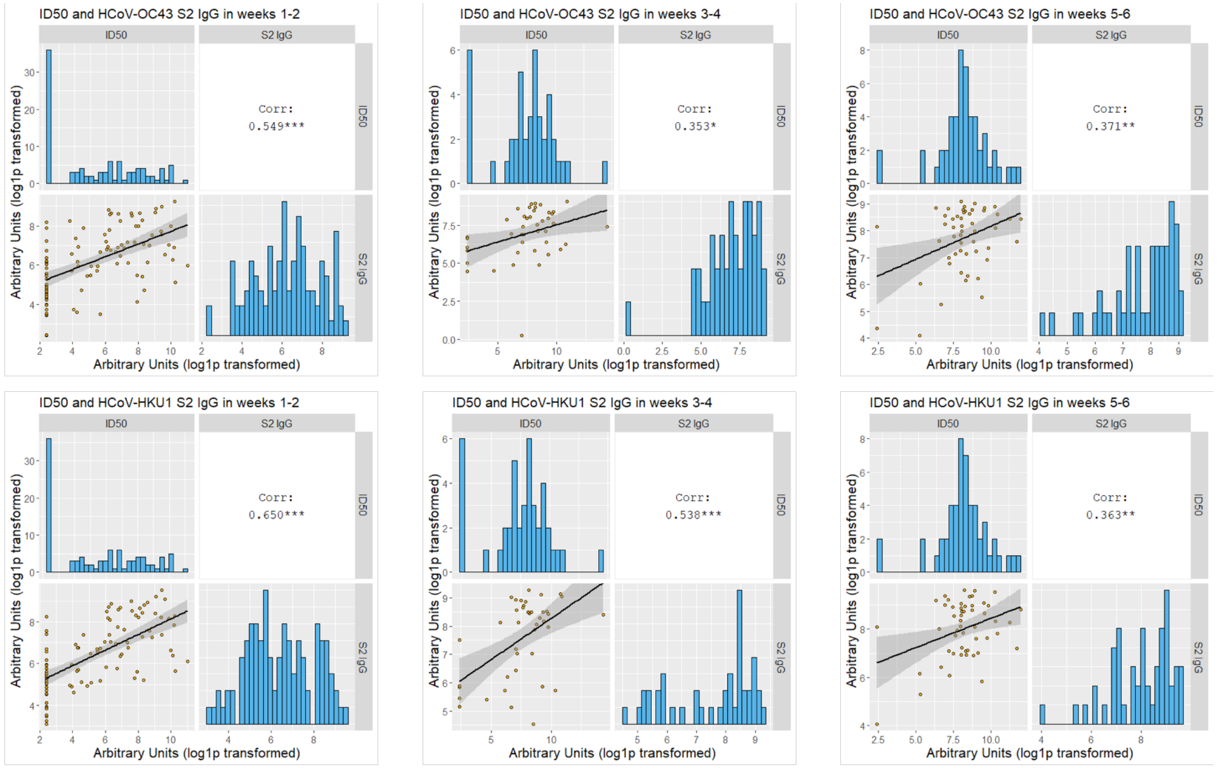

Footnote to Supplementary Figure 7: HCoV-HKU1 and -OC43 S2 IgG correlation with neutralization is temporary. Sera of COVID-19 patients, collected at the indicated timepoints from symptoms onset, were measured by LV-based neutralization assay and the LIPS assay indicated in grey labels above each row/column. Boxes under the diagonal show each correlation plot of the ID50 reciprocal and arbitrary units after log10 conversion. Dots correspond to individual measurements, the black line represents the regression line and the grey area its 95%CI. Boxes on the diagonal show as histograms the distribution of values in each assay. Boxes above the diagonal show the corresponding Pearson correlation analysis coefficients. Asterisks correspond to the following p values: *** p ≤ 0.001; ** p ≤ 0.01; * p ≤ 0.05. Source data are provided as a Source Data file.

Supplementary Figure 8

a

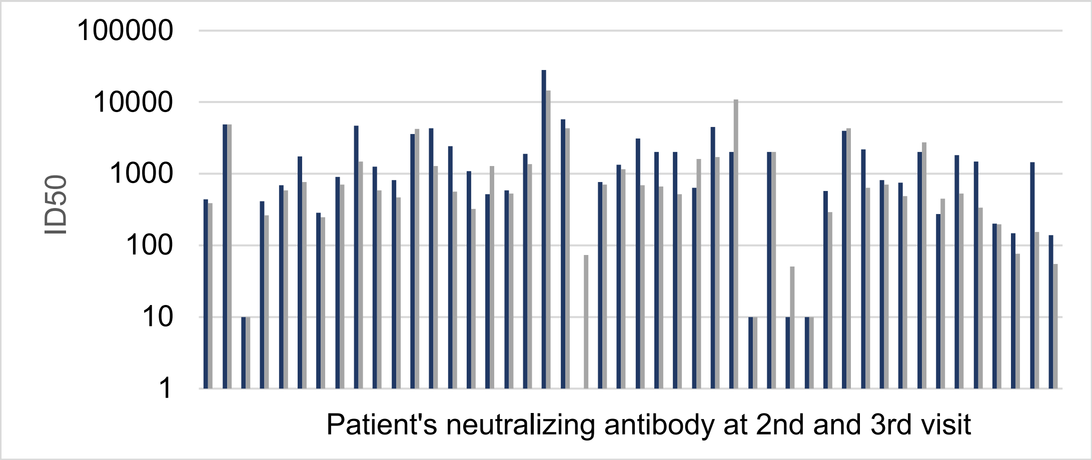

b

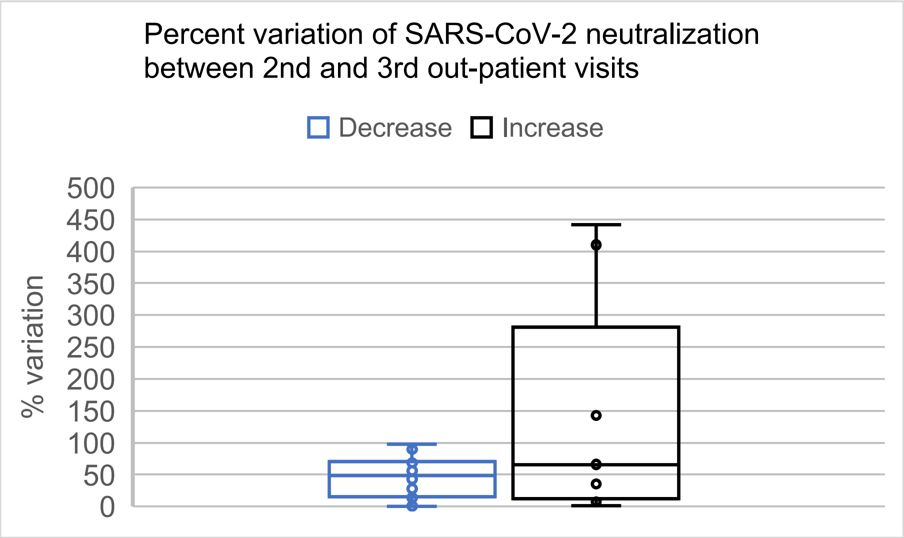

No. of individuals	34	9
Median % variation	48,78	65,90
Range (min-max)	0,3-98,13	1,29-441,92
Standard deviation	28,01	170,41
No. with variation >30%	22	6

Footnote to Supplementary Figure 8: Variations of the SARS-CoV-2 neutralizing titers between the 2nd and 3rd out-patient visit. (a) the inhibitory serum dilution (ID50) at the 2nd and 3rd visit (dark and light grey bars, respectively), and (b) the percent variation of the ID50 of each of the 43 patients tested for nAbs. The 3rd visit occurred between September 22nd and November 6th 2020, when the Rt in the same geographical area of the San Raffaele Hospital, the Lombardy region, ramped-up from 0.86 (CI 0.73-1.01) at the end of September to 1.61 (CI 1.08-2.33) in the first week of November. (http://www.salute.gov.it/portale/nuovocoronavirus/dettaglioNotizieNuovoCoronavirus.jsp?lingua=italiano&menu=notizie&p=dalministero&id=5093). Source data are provided as a Source Data file.

Supplementary Figure 9

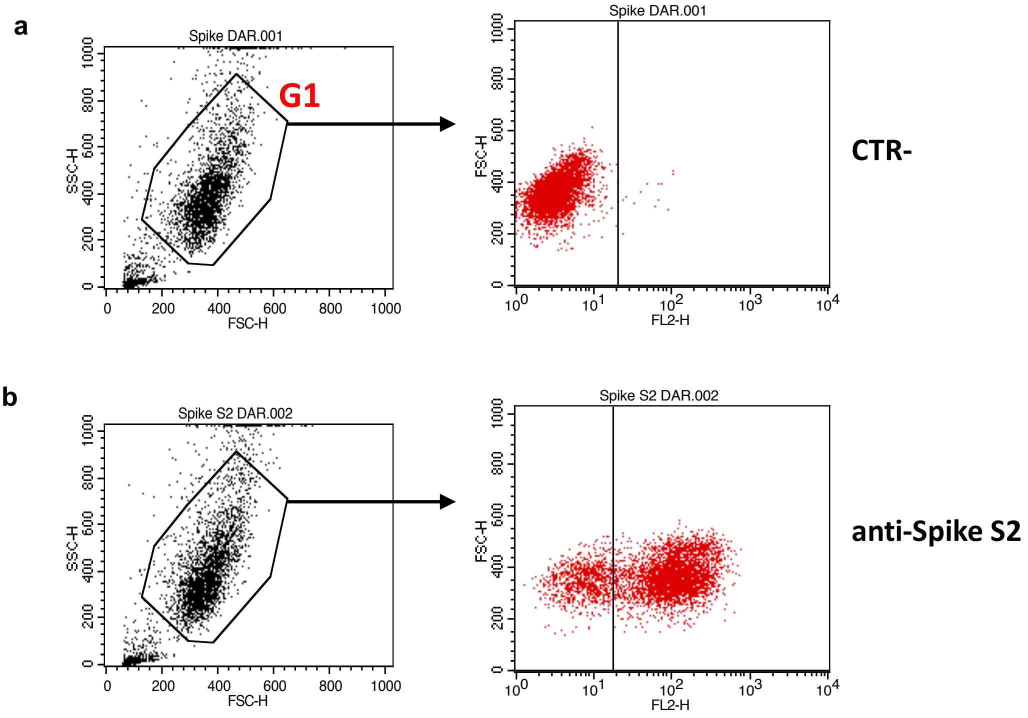

Footnote to Supplementary Figure 9: Gating strategy used for FACS analysis to detect SARS-CoV-2 spike. Forward (FSC) and side scatter (SSC) gating (G1) was used to identify the cells of interest, while removing debris. Lenti-X cells transfected with pSpike were gated and analyzed for Spike expression using a primary rabbit-anti-Spike S2 antibody (Sino Biological, Catalog Number: 40590-T62) followed by a secondary PE donkey anti-rabbit antibody (Biolegend, Catalog Number: 406421). Staining with secondary antibody only was used as negative control (CTR-, in panels a) to set the quadrant and quantify the percentage of Spike-expressing cells (in panels b).

Supplementary Figure 10

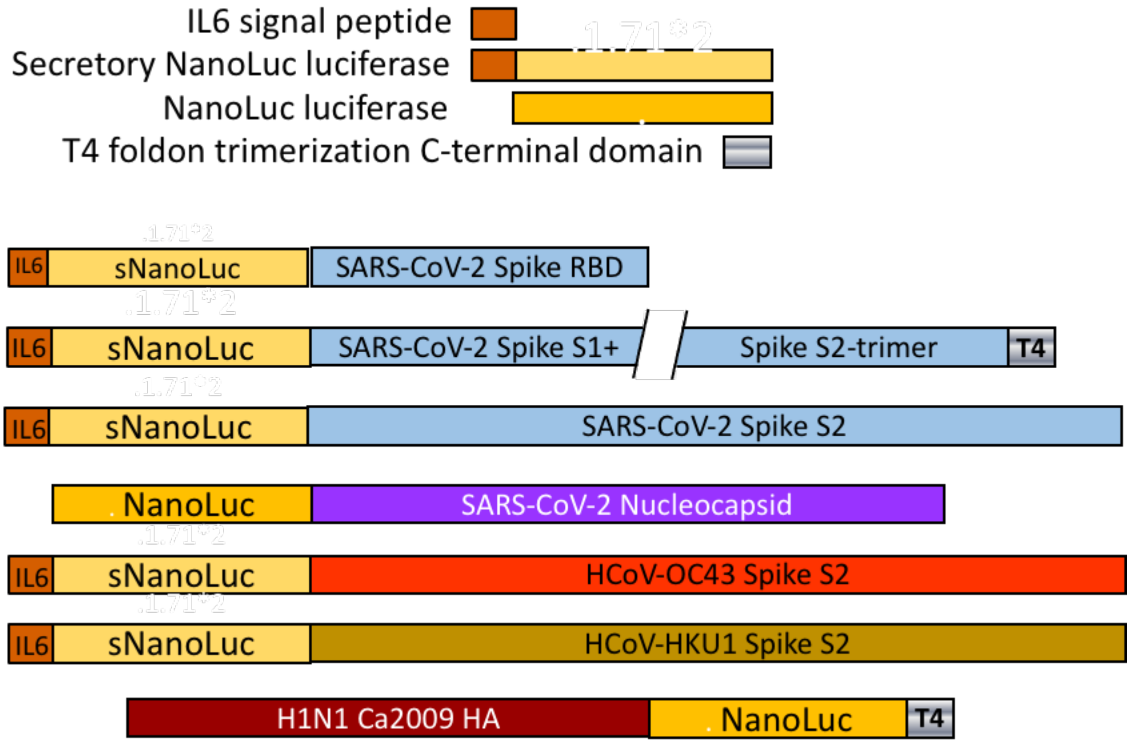

Footnote to Supplementary Figure 10. Schematic representation of plasmids used for the LIPS. The recombinant nanoluciferase (sNanoLuc) tagged antigens used in this study are shown. Serial Cloner 2.6.1 for virtual design of recombinant antigens plasmid clones. See Methods for details on construction.

Supplementary Tables

Supplementary Table 1: Laboratory values at first Biobank blood sampling of the COVID-19 study population (No. of patients = 162).

	COVID-19 patients	Missing data
Median days (95%CI) from symptoms onset to first sampling for Biobank	11.5 (7-18)	0
Laboratory values at time of first blood sampling (Median, 95%CI):		
- White Blood Cells, x10^9/L	6.9 (5.2-9.8)	17
- Lymphocyte (Ly) count, x10^9/L	1.1 (0.7-1.5)	25
- Neutrophil (N) count, x10^9/L	4.8 (3.5-7.5)	25
- Monocyte count, x10^9/L	0.5 (0.4-0.7)	25
- N/Ly ratio	4.67 (2.55-8.75)	25
- Haemoglobin, g/dL	13.1 (11.6-14.6)	17
- Platelet count, x10^9/L	236 (182-323)	17
- Bilirubin total, mg/dL	0.56 (0.37-0.86)	45
- ALT, U/L	41 (23.5-66.5)	35
- AST, U/L	44 (31-60)	34
- Creatinine, mg/dL	0.97 (0.77-1.34)	23
- LDH, U/L	355 (273-428)	39
- CRP, mg/L	54.6 (18.75-118.1)	19
- D-Dimer, µg/mL	1.11 (0.53-2.45)	104
- IL-6, pg/mL	33 (16.7-81)	117
- Ferritin, ng/mL	1222 (597-1701)	103

Footnote to Supplementary Table 1: ALT: Alanine Amino Transferase; AST: Aspartate Amino Transaminase; LDH: Lactate dehydrogenase; CRP: C-reactive protein.

Supplementary Table 2: SARS-CoV-2 Antibody responses according to week from symptoms onset.

	Weeks after symptoms onset					
	**1-2**	**3-4**	**5-8**	**9-16**	**17-36**	**Overall**
Samples tested (No.)	101	43	76	76	66	362
Neutralization of SARS-CoV-2						
Mean (SD)	3500 (8600)	45300 (252000)	9950 (24400)	2160 (3910)	1460 (1910)	9190 (87900)
Median	238	2640	3500	869	660	1080
[Min, Max]	[10, 61500]	[10, 1660000]	[10, 161000]	[10, 28000]	[10, 10900]	[10, 1660000]
Antibody binding to SARS-CoV-2						
IgG_RBD						
Mean (SD)	13.0 (29.4)	165 (296)	2670 (5710)	4360 (7570)	6100 (6640)	2610 (5660)
Median	1,4	43	1060	2010	3830	333
[Min, Max]	[0.00296, 163]	[0.00859, 1130]	[0.116, 46500]	[0.0453, 36800]	[0.0171, 31500]	[0.00296, 46500]
IgG_S1S2						
Mean (SD)	37.5 (50.6)	1370 (4040)	8850 (8960)	8080 (8660)	7310 (6970)	5060 (7580)
Median	8,66	82,3	6420	5350	6650	1080
[Min, Max]	[0.0228, 258]	[0.379, 23400]	[0.160, 46900]	[6.65, 33400]	[0.112, 33400]	[0.0228, 46900]
IgG_S2						
Mean (SD)	349 (1760)	818 (3030)	1550 (4050)	1640 (1670)	3550 (3930)	1510 (3140)
Median	7,81	159	429	1090	2740	312
[Min, Max]	[0.00373, 16000]	[0.00885, 18800]	[0.0289, 31000]	[0.0159, 6400]	[0.0169, 25600]	[0.00373, 31000]
IgG_NP						
Mean (SD)	20.3 (19.7)	31.0 (19.8)	49.3 (18.9)	NA	NA	28.3 (22.4)
Median	12,4	35,7	53,1			29,4
[Min, Max]	[0.237, 65.9]	0.313, 65.6]	[0.443, 75.4]			[0.237, 75.4]
missing	1 (1%)	4 (9.3%)	43 (56.6%)	100%	100%	190 (52.5%)
Antibody binding to other Viruses						
IgG_OC43 S2						
Mean (SD)	1430 (2040)	2310 (2370)	3490 (2430)	2610 (2070)	1630 (1190)	2290 (2220)
Median	492	1220	3410	2350	1320	1440
[Min, Max]	[10.2, 10200]	[0.307, 8410]	[60.1, 8780]	[29.8, 8340]	[57.6, 4420]	[0.307, 10200]
missing	0%	0%	0%	0%	18 (27.3%)	18 (5.0%)
IgG_HKU1 S2						
Mean (SD)	1760 (2550)	3600 (3030)	4650 (4060)	3580 (3020)	2120 (1780)	3080 (3210)
Median	587	3410	3240	2910	1590	1930
[Min, Max]	[21.4, 13500]	[92.8, 10500]	[56.8, 15100]	[2.73, 12800]	[108, 7310]	[2.73, 15100]
missing	0%	0%	0%	0%	18 (27.3%)	18 (5.0%)
IgG_FLU HA						
Mean (SD)	18600 (25600)	12500 (20900)	13300 (15100)	NA	NA	16200 (23000)
Median	7040	4750	6940			6890
[Min, Max]	[72.6, 108000]	[172, 117000]	[311, 58200]			[72.6, 117000]
missing	1 (1%)	4 (9.3%)	43 (56.6%)	100%	100%	190 (52.5%)
IgM_FLU HA						
Mean (SD)	9.34 (15.4)	11.0 (17.7)	14.0 (23.3)	NA	NA	10.6 (17.6)
Median	2,82	3,52	2,97			3,12
[Min, Max]	[0.328, 92.5]	[0.159, 90.8]	[0.377, 91.4]			[0.159, 92.5]
missing	1 (1%)	4 (9.3%)	44 (57.9%)	100%	100%	191 (52.8%)
Days post symptoms Median [Min, Max]	8.00 [1, 14]	18.0 [15, 28]	39.0 [30, 54]	95.0 [57, 112]	204 [114, 250]	39.0 [1, 250]
Age years Median [Min, Max]	63.0 [34, 94]	63.0 [34, 88]	61.5 [19, 87]	59.5 [26, 87]	61.0 [37, 87]	62.0 [19, 94]
Sex Male (No.)	67 (66.3%)	31 (72.1%)	48 (63.2%)	49 (64.5%)	41 (62.1%)	236 (65.2%)
Sample Category No.						
In-hospital visit	101 (100%)	39 (90.7%)	10 (13.2%)	0 (0%)	0 (0%)	150 (41.4%)
1st follow-up visit	0 (0%)	4 (9.3%)	66 (86.8%)	17 (22.4%)	0 (0%)	87 (24.0%)
2nd follow-up visit	0 (0%)	0 (0%)	0 (0%)	59 (77.6%)	18 (27.3%)	77 (21.3%)
3rd follow-up visit	0 (0%)	0 (0%)	0 (0%)	0 (0%)	43 (65.2%)	43 (11.9%)
4th follow-up visit	0 (0%)	0 (0%)	0 (0%)	0 (0%)	4 (6.1%)	4 (1.1%)
5th follow-up visit	0 (0%)	0 (0%)	0 (0%)	0 (0%)	1 (1.5%)	1 (0.3%)

Footnote to Supplementary Table 2: No. = number. Neutralization is expressed as the inverse of the serum dilution at which the ID50 was obtained in the LV-based SARS-CoV-2 neutralization assay. A value of 10 was ascribed to the serum that displayed absence of neutralization at the first dilution used (1/40) for the assay. Antibody binding is expressed as arbitrary units. Sample category shows the number and percent of patient's sera tested at each visit according to time (in week intervals) from symptoms onset as depicted in Figure 2. NA= not applicable.

Supplementary Table 3: Characteristics of non-hospitalized COVID-19 patients.

	**Non-hospitalized COVID-19 patients (N=26)**
Age, years	49.5 (46-62)
Sex Male	38.5%
Ethnicity:	
o Caucasian	84.6%
o Hispanic	15.4%
o Asian	0
o African	0
Co-morbidities:	
o Hypertension	11.5%
o CAD	7.7%
o Diabetes	7.7%
o COPD	0%
o CKD	3.8%
o Cancer	0%
o Neurodegenerative disease	0%
Number of co-morbidities	
o None	80.8%
o 1	11.5%
o 2	3.8%
o 3	3.8%
o 4	0
Body Mass Index:	
o <25	31.8%
o 25-30	40.9%
o >30	27.3%
Median days (95%CI) from symptoms onset:	
- to admission at Emergency Department	5.5 (3-10)
- to first blood sampling for Biobank	21.5 (5-37)
Symptoms at disease onset:	
- general	
o fever	68%
o headache	36%
o fatigue/malaise	60%
o myalgia/arthralgia	36%
- respiratory	
o cough	60%
o dyspnea	28%
o sore throat	20%
o chest pain	24%
- gastrointestinal	
o diarrhea	40%
o vomiting/nausea	12%
o abdominal pain	12%
- others	
o conjunctivitis	12%
o hypo/anosmia	28%
o hypo/dysgeusia	40%
o skin rash	1.3%
Median days from symptoms to negative RT-PCR swab (95%CI)	33 (31-35)
Median follow up days (95%CI)	97 (89-104)

Footnote to Supplementary Table 3: abbreviations are as in Table 2.

Supplementary Table 4: Characteristics of COVID-19 patients grouped according to their SARS-CoV-2 neutralizing antibody response.

Characteristics	SARS-CoV-2 neutralizing Ab score				Statistics
	Negative (N=43 patients)	M	Positive (N=107 patients)	M	p Value
Median age, years (95%CI)	74 (59-79)	0	62 (51-70)	0	0,003
Co-morbidities:					
○ Hypertension	26 (60.5%)		45 (42.1%)		0,048
○ CAD	8 (18.6%)		13 (12.1%)		0,308
○ Diabetes	10 (23.3%)	0	30 (28%)	0	0,684
○ COPD	5 (11.6%)		1 (0.9%)		0,008
○ CKD	15 (34.9%)		9 (8.4%)		<0.001
○ Cancer	10 (23.3%)		7 (6.5%)		0,008
○ Neurodegenerative disease	3 (7%)		1 (1.9%)		0,142
Number of co-morbidities					
○ None	10 (23.3%)		48 (44.9%)		
○ 1	6 (14%)	0	22 (20.6%)	0	0,005
○ 2	14 (32.6%)		28 (26.2%)		
○ 3	9 (20.9%)		7 (6.5%)		
○ 4	4 (9.3%)		2 (1.9%)		
Body Mass Index					0,012
<25	15 (44.1%)	9	19 (18.6%)	5	
25-30	11 (32.4%)		45 (44.1%)		
>30	8 (23.5%)		38 (37.3%)		
Symptoms at disease onset:		1		4	
- general					
○ fever	71,40%		91,30%		0,004
○ headache	21,40%		25,20%		0,541
○ fatigue/malaise	28,60%		67%		<0.001
○ myalgia/arthralgia	26,20%		35,90%		0,331
- respiratory					
○ cough	35,70%		71,80%		<0.001
○ dyspnea	47,60%		79,60%		<0.001
○ sore throat	11,90%		17,50%		0,464
○ chest pain	14,30%		26,20%		0,133
- gastrointestinal					
○ diarrhea	16,70%		31,10%		0,099
○ vomiting/nausea	9,50%		14,60%		0,589
○ abdominal pain	11,90%		7,80%		0,523
- others					
○ conjunctivitis	4,80%		18,40%		0,038
○ hypo/anosmia	16,70%		46,60%		0,001
○ hypo/dysgeusia	21,50%		50,50%		0,002
○ skin rash	7,10%		3,90%		0,413
Median days (95%CI) from symptoms onset:					
- to admission	5 (3-12)	0	10 (7-12)	0	<0.001
- to blood sampling for Biobank	6 (4-12)		13 (9-16)		<0.001
Admitted to the hospital	43/45 (95.5%)		107/117 (91.4%)		
- Discharged	5/43 (11.6%)		11/107 (10.3%)		
- Hospitalized					
○ ≤7days	6/43 (14%)		19/107 (17.8%)		
○ >7days	16/43 (37.2%)	0	49/107 (45.8%)	0	<0.001
○ deceased	15/43 (34.9%)		3/107 (2.8%)		
○ in need of ICU, recovered	1/43 (2.3%)		14/107 (13.1%)		
○ in need of ICU, deceased	0/43 (0%)		11/107 (7.3%)		
Median days of hospital stay for 134 patients (95%CI)	8 (4-24)		13 (6-21)		0,317
Median days from symptoms to negative RT-PCR swab (95%CI)	46 (38-54)	2	40 (37-43)	5	0,041
Median follow up days (95%CI)	194 (99-289)	0	203 (198-208)	0	0,521
Laboratory values at time of first blood sampling:					
- White Blood Cells, $x10^9$/L	6.2 (4.7-8.1)	5	7.2 (5.5-10.3)	12	0,028
- Lymphocyte (Ly) count, $x10^9$/L	0.95 (0.5-1.5)	9	1.2 (0.8-1.5)	16	0,088
- Neutrophil (N) count, $x10^9$/L	4.3 (2.65-6.75)	9	4.9 (3.7-7.8)	16	0,085
- Monocyte count, $x10^9$/L	0.4 (0.3-0.6)	9	0.6 (0.4-0.8)	16	0,009
- N/Ly ratio	4.2 (2.46-8.25)	9	4.92 (2.55-9.04)	16	0,986
- Haemoglobin, g/dL	11.95 (9.72-14.05)	5	13.5 (12.1-14.8)	12	0,001
- Platelet count, $x10^9$/L	187 (109-234)	5	256 (199-355)	12	<0.001
- Bilirubin total, mg/dL	0.44 (0.36-0.72)	13	0.65 (0.38-0.98)	32	0,053
- ALT, U/L	30 (18.5-34.5)	12	47 (26-75)	23	<0.001
- AST, U/L	36 (26-53)	12	46 (33-64)	22	0,045
- Creatinine, mg/dL	1.22 (0.87-1.81)	7	0.95 (0.76-1.24)	16	0,002
- LDH, U/L	282 (232-409)	12	368.5 (294-441)	27	0,007
- CRP, mg/L	46.4 (20.4-99)	7	62.6 (18.1-128.1)	12	0,417

Footnote to Supplementary Table 4: The median BMI is 25.9 (IQR 23-29.7) for the neutralizing antibody negative group and 28.3 (IQR 25.4-32.3) for the neutralizing antibody positive group of patients (p= 0.018). Chi-square or Fischer's exact test were used to compare categorical variables. Wilcoxon rank sum test was used to compare continuous variables. A two-sided P Value was reported. Abbreviations used are: M = missing data. Intensive Care Unit (ICU) Coronary Artery Diseases (CAD), Chronic obstructive pulmonary disease (COPD), Chronic Kidney Disease (CKD), Neurodegenerative disease (ND); Lactate dehydrogenase (LDH), C-reactive protein (CRP), Alanine Amino Transferase (ALT), Aspartate Amino Transaminase (AST), Lactate dehydrogenase (LDH), C-reactive protein (CRP).

Supplementary Table 5: COVID-BioB study team and collaborators.

Affiliation is IRCCS Ospedale San Raffaele, Milan, Italy.

Name	Affiliation
Giovanni Landoni	Università Vita-Salute San Raffaele
Alberto Zangrillo	Università Vita-Salute San Raffaele
Angelo AM Manfredi	Autoimmunity and Vascular Inflammation Unit
Antonella Castagna	Department of Infectious Diseases
Paolo Scarpellini	Department of Infectious Diseases
Paola Cinque	Department of Infectious Diseases
Andrea Poli	Department of Infectious Diseases
Laura Galli	Department of Infectious Diseases
Roberta Caccia	Department of Infectious Diseases
Patrizia Rovere Querini	Department of Internal Medicine
Elena Cantarelli	Molecular Hematology Unit
Michela Grossi	Molecular Hematology Unit
Chiara Bonini	Division of Immunology, Transplantation and Infectious Diseases
Mauro Malnati	Division of Immunology, Transplantation and Infectious Diseases
Massimo Clementi	Laboratory of Microbiology and Virology
Nicasio Mancini	Laboratory of Microbiology and Virology
Elisa Vicenzi	Viral Pathogens and Biosafety Unit
Guido Poli	Viral Pathogens and Biosafety Unit
