# Supplementary material for: Neutralizing antibody responses to SARS-CoV-2 in symptomatic COVID-19 is persistent and critical for survival
Source: Nat Commun. 2021 May 11;12:2670. doi: 10.1038/s41467-021-22958-8 (PMC8113594; doi:10.1038/s41467-021-22958-8)
Supplement: Supplementary file 3 — Reporting Summary [file 41467_2021_22958_MOESM3_ESM.pdf]

nature research

Corresponding author(s): Gabriella SCARLATTI.
Last updated by author(s): Mar 30, 2021

Reporting Summary

Nature Research wishes to improve the reproducibility of the work that we publish. This form provides structure for consistency and transparency in reporting. For further information on Nature Research policies, see our Editorial Policies and the Editorial Policy Checklist.

Statistics

For all statistical analyses, confirm that the following items are present in the figure legend, table legend, main text, or Methods section.

n/a	Confirmed	
☐	☒	The exact sample size (*n*) for each experimental group/condition, given as a discrete number and unit of measurement
☐	☒	A statement on whether measurements were taken from distinct samples or whether the same sample was measured repeatedly
☐	☒	The statistical test(s) used AND whether they are one- or two-sided<br>*Only common tests should be described solely by name; describe more complex techniques in the Methods section.*
☐	☒	A description of all covariates tested
☐	☒	A description of any assumptions or corrections, such as tests of normality and adjustment for multiple comparisons
☐	☒	A full description of the statistical parameters including central tendency (e.g. means) or other basic estimates (e.g. regression coefficient) AND variation (e.g. standard deviation) or associated estimates of uncertainty (e.g. confidence intervals)
☐	☒	For null hypothesis testing, the test statistic (e.g. *F*, *t*, *r*) with confidence intervals, effect sizes, degrees of freedom and *P* value noted<br>*Give P values as exact values whenever suitable.*
☒	☐	For Bayesian analysis, information on the choice of priors and Markov chain Monte Carlo settings
☒	☐	For hierarchical and complex designs, identification of the appropriate level for tests and full reporting of outcomes
☐	☒	Estimates of effect sizes (e.g. Cohen's *d*, Pearson's *r*), indicating how they were calculated

Our web collection on statistics for biologists contains articles on many of the points above.

Software and code

Policy information about availability of computer code

Data collection	Clinical data collection and management performed with a eCRF in MySQL (V5.7.14) on Apache Tomcat (V2.4.23) platform running on windows sever 2012 R2. Microsoft Excel versions used for data collection are Macv16.43 and Office365 2020. MikroWin version 5.22 was used for collection of luminometer data acquired with Berthold Centro XS3 luminometer for LIPS. Luminometer data of neutralization assays were acquired with Mithras LB940 Berthold, using Microsoft Excel for Mac V16.43. Serial Cloner 2.6.1 for virtual design of recombinant antigens plasmid clones. Beckman Coulter Navios and FACSCalibur were used for flowctyometric acquisitions.
Data analysis	Statistical analysis and data visualization was performed using SPSS 24 (SPSS Inc./IBM) and the R software version 3.4.3. FACS data were analyzed by BD CellQuest Pro software version 6.0 and FlowJo version 8.8.7 (Tree Star).

For manuscripts utilizing custom algorithms or software that are central to the research but not yet described in published literature, software must be made available to editors and reviewers. We strongly encourage code deposition in a community repository (e.g. GitHub). See the Nature Research guidelines for submitting code & software for further information.

Data

Policy information about availability of data

All manuscripts must include a data availability statement. This statement should provide the following information, where applicable:

- Accession codes, unique identifiers, or web links for publicly available datasets
- A list of figures that have associated raw data
- A description of any restrictions on data availability

The data that support the findings of this study are available from the corresponding author upon reasonable request. Source data are provided with this paper.

Field-specific reporting

Please select the one below that is the best fit for your research. If you are not sure, read the appropriate sections before making your selection.

☒ Life sciences ☐ Behavioural & social sciences ☐ Ecological, evolutionary & environmental sciences

For a reference copy of the document with all sections, see nature.com/documents/nr-reporting-summary-flat.pdf

Life sciences study design

All studies must disclose on these points even when the disclosure is negative.

Sample size	Finally 162 patients were included in the Immunological study of this paper, and the antibody levels tested in total 362 sera. The 162 patients of the Immunogical sub-study (called ImmCOVID-Role of the immune response in the infection with SARS-CoV-2 and in the pathogenesis of COVID-19) represent the overall COVID-19 BioB cohort recruited during the period from February 25th to end of April 2020, at the Ospedale San Raffaele in Milan, Italy, in terms of % hospitalization, co-morbidities, need of Intensive care and fatal outcome. First dedicated blood sample (serum) was available from the COVID.BioB cohort from 150 patients at the in-hospital stay (at emergency department or ward), and from 12 at the out-patient clinic visit. Recovered patients returning for follow-up visits within COVID-BioB were included for the observational progressive immunological study: 87 patients attended also the first follow-up visit (at 1 month after discharge), 77 attended also the second visit (3 months after discharge) and 46 also the third visit (6 months after discharge). Follow-up of the immunological study was closed on November 25th 2020. No other criteria were used to determine the sample size. Details are in Table 1 Clinical management and serum samples availability of the COVID-19 study patients. Missing clinical data and laboratory values at the in-hospital visit of the immunological study cohort are detailed in Table 2 and Supplementary Table 1.
Data exclusions	Exclusion criteria of a study subject for the immunological data analysis were 1) the absence of a confirmed SARS-COV-2 infection (excluded 17 patients, and used as negative controls), and 2) for the neutralization assay results, to exclude any potential unspecific inhibition of the serum each serum was tested in duplicate at 1/40 dilution with LV-Luc/VSV.G in the neutralization assay. Serum with >50% VSV inhibition compared to virus control was discarded from any further analysis as it would not allow for a correct determination of the SARS-CoV-2 specific inhibition. We had to exclude 5 patients. Overall, analysis of the neutralizing antibody levels was performed with 362 sera of 162 SARS-CoV-2 confirmed cases.
Replication	Replication of the sample collection was not relevant because the study intended to collect only one serum sample at each time-point of the study.<br>LIPS and neutralization assays were optimized, and key parameters defined to control for assay variability and reproducibility. The validation of the LIPS assay is published in Secchi et al JCI 2020. While the parameters used for the neutralization assay are detailed in this manuscript in the Method section: Lentiviral vector-based SARS-CoV-2 neutralization assay. Antibody determinations were performed as end-point dilutions and in duplicates. In specific: A test was valid if the percent Coefficient Variation RLUs of the virus control wells was ≤30%, and the value of both positive control sera was within a 2-fold range of the median inter-assay ID50 value. A test sample result was discarded and the serum re-tested when the percent difference between duplicate wells was >30% for those sample dilutions that yielded at least 40% inhibition.
Randomization	The COVID-19 Biob clinical trial has no randomization criteria as the study is observational and participants did not undergo any experimental treatment protocol.<br><br>For the immunological antibody evaluation study: Time-to-event was calculated from the date of symptoms onset to the date of death, or of last follow up visit, whichever occurred first. Survival was estimated according to Kaplan–Meier. Association between antibody positivity and time to death was calculated using univariate and multivariate Cox proportional hazards models. The effect was reported as hazard ratio (HR) with the corresponding 95% CI, estimated using the Wald approximation. All survival analysis and association were stratified according to time from the onset of symptoms to blood sampling (weeks 1, 2, 3, ≥ 4). Two-tailed p values are reported, with p value <0.05 indicating statistical significance. All confidence intervals are two-sided and not adjusted for multiple testing.
Blinding	Data were de-identified and analysed retrospectively. Investigators performing the serum antibody binding and neutralization assays were blinded for the clinical and laboratory data of the patients. All the studies performed on biological samples were made by investigator blinded to clinical data, group collection and analysis.

Reporting for specific materials, systems and methods

We require information from authors about some types of materials, experimental systems and methods used in many studies. Here, indicate whether each material, system or method listed is relevant to your study. If you are not sure if a list item applies to your research, read the appropriate section before selecting a response.

Materials & experimental systems

n/a	Involved in the study	
[ ]	[x]	Antibodies
[ ]	[x]	Eukaryotic cell lines
[x]	[ ]	Palaeontology and archaeology
[x]	[ ]	Animals and other organisms
[ ]	[x]	Human research participants
[ ]	[x]	Clinical data
[x]	[ ]	Dual use research of concern

Methods

n/a	Involved in the study	
[x]	[ ]	ChIP-seq
[ ]	[x]	Flow cytometry
[x]	[ ]	MRI-based neuroimaging

Antibodies

Antibodies used

Primary antibodies: anti-human ACE2 (Millipore, Billerica, MA; Cat. MAB5676); polyclonal anti-Spike SARS-CoV-2 (Sino Biological, Prodotti Gianni, Italy, Cat. 40590-T62); anti-Gag antibody (NIH Repository Reagents, Cat. #4250).
Secondary antibodies: Phycoerythrin conjugated goat anti-mouse secondary antibody (SouthernBiotech, USA; Cat. 1030-09); donkey anti-rabbit PE (Biolegend, San Diego, CA, USA; Cat. 406421).

Validation

Validation statements for the antibodies that are commercially available are:
-Anti-human ACE2 (Millipore, cod. MAB5676): mouse monoclonal obtained after immunization with recombinant human ACE2. Indicated specificity for ACE2 ([Angiotensin-converting enzyme 2, SARS receptor). https://www.merckmillipore.com/IT/it/product/Anti-ACE2-Antibody,MM_NF-MAB5676

-Polyclonal anti-Spike SARS-CoV-2 (Sino Biological, Prodotti Gianni, Italy, Cat. 40590-T62): rabbit polyclonal antibody obtained after immunization with recombinant SARS-CoV-2/2019-nCoV Spike/S2 Protein. The specific IgG were purified by SARS-CoV-2/2019-nCoV Spike/S2 affinity chromatography. Specific for SARS-CoV-2 Spike protein in Western Blot. https://www.sinobiological.com/antibodies/cov-spike-40590-t62

-Anti-Gag antibody (NIH Repository Reagents, USA, Cat. #4250): rabbit polyclonal anti-HIV-1 p24 Gag protein obtained after immunization of rabbits with HIV-1 SF2 p24 expressed in yeast. Titer indicated for use is >50,000 in WB and >100,000 in ELISA. https://www.hivreagentprogram.org/Catalog/HRPPolyclonalAntiserum/ARP-4250.aspx

Eukaryotic cell lines

Policy information about cell lines

Cell line source(s)

VEROE6 (African green monkeys, kidney), Caco2 (human, colon), Calu3 (human, lung), HUH7 (human, liver), and 293T (human, kidney) cell lines (from ATCC); Expi293F™ cells (Expi293™ Expression System, Thermo Fisher Scientific Life Technologies, Carlsbad, CA, USA); 293T Lenti-X (Clontech, CA, USA).

Authentication

No authentication was performed.

Mycoplasma contamination

VeroE6 and 293T Lenti-X were tested for mycoplasma contamination, while the others were not. The VEROE6 and 293T Lenti-X cell lines tested negative with MycoSPY® Master Mix - PCR Mycoplasma Test Kit della Biontex (link: https://www.biontex.com/en/shop/mycospy-mm.html).

Commonly misidentified lines
(See ICLAC register)

No commonly misidentified cell lines were used.

Human research participants

Policy information about studies involving human research participants

Population characteristics	COVID-19 patients attending the Hospital San Raffaele were predominantly Caucasian, male, had a median age of 63 years and approximately two third had one or more co-morbidities as described in the manuscript. This reflect the overall italian epidemic during the period February - June 2020.
Recruitment	Patients, age 18 or older, were recruited from the COVID-BioB protocol study and serum biobanked. This included: Patients admitted to hospital with biological samples positive for SARS-CoV-2; Patients admitted to hospital with negative test but clinical and radiological characteristics highly suggestive of SARS-CoV-2 disease; Patients discharged from emergency department with biological samples positive for SARS-CoV-2. Recovered patients were suggested to attend the follow-up visit a Hospital San Raffaele. At follow-up blood (serum) was collected for the study. The Immunological (ImmCOVID) follow-up study was closed November 25th, 2020.<br>Patients and doctor during follow-up were blinded to antibodies results and all the subject available were included in the analysis. The patient retention during follow-up could be influenced by the severity of disease at the onset or the persistence of symptoms (Long-Covid). These could have selected a population with more severe disease during the follow up.
Ethics oversight	Institutional Review Board at IRCCS Ospedale San Raffaele authorized the COVID-Biob clinical trial with collection of biologial material (protocol number 34/INT/2020), and the related Immunological study called ImmCOVID (protocol number 68/INT/2020).

Note that full information on the approval of the study protocol must also be provided in the manuscript.

Clinical data

Policy information about clinical studies

All manuscripts should comply with the ICMJEguidelines for publication of clinical research and a completedCONSORT checklist must be included with all submissions.

Clinical trial registration	ClinicalTrialsgov-NCT04318366
Study protocol	COVID-19 Patients Characterization, Biobank, Treatment Response and Outcome Predictor (COVID-BioB). The protocol, which is in italian language, can be requested to the principal investigator (Fabio Ciceri).
Data collection	Observational cohort with non-probability sampling method. Data of COVID-19 clinical-biological cohort study (COVID-BioB) were collected at the IRCCS San Raffaele Hospital. Data were collected from medical chart review or directly by patient interview and entered in a dedicated electronic case record form (eCRF). Data were crosschecked and verified by data managers and clinicians for accuracy.<br>COVID-Biob observational study started on February 25th 2020, related biobanking on March 8th, and is still ongoing. Samples for the immunological study were obtained from patients hospitalized and followed from March till November 2020.
Outcomes	COVID-BioB goal was the inclusion of at least 1000 COVID-19 patients with collection of biological materials (blood, serum, swabs primarily) for subsequent use for substudies.

Flow Cytometry

Plots

Confirm that:

- [ ] The axis labels state the marker and fluorochrome used (e.g. CD4-FITC).
- [x] The axis scales are clearly visible. Include numbers along axes only for bottom left plot of group (a 'group' is an analysis of identical markers).
- [x] All plots are contour plots with outliers or pseudocolor plots.
- [x] A numerical value for number of cells or percentage (with statistics) is provided.

Methodology

Sample preparation	1) To verify the expression of ACE2 on the cell lines 200.000 cells were used.<br>2) To veridy expression of the SARS-CoV-2 spike on Lenti-X 293T cells 500.000 cells were used.
Instrument	1) Navios flow cytometer (Beckman Coulter, Inc., CA, USA)<br>2) FACSCalibur flow cytometer (BD Biosciences, Milan, Italy)
Software	1) FlowJo version 8.8.7 (Tree Star)<br>2) CellQuest Pro software (BD Biosciences)
Cell population abundance	na

Gating strategy	Both gating strategies are described in supplementary informations in figure 2 (ACE 2 expression) and figure 9 (SARS-CoV-2 spike expression on LentiX 293T cells).

☒ Tick this box to confirm that a figure exemplifying the gating strategy is provided in the Supplementary Information.
